# Brief intervention for hazardous drinking delivered using text messaging: a pilot randomised controlled trial from Goa, India

**DOI:** 10.1017/S1368980022000313

**Published:** 2022-05

**Authors:** Abhijit Nadkarni, Danielle Fernandes, Urvita Bhatia, Richard Velleman, Ethel D’souza, Joseline D’souza, Grace Marimilha Pacheco, Seema Sambari

**Affiliations:** 1 Centre for Global Mental Health, London School of Hygiene & Tropical Medicine, London, UK; 2 Addictions Research Group, House No. 451 (168), Bhatkar Waddo, Socorro, Porvorim, Bardez, Sangath, Goa 403501, India; 3 Department of Psychology, University of Bath, Bath, UK

**Keywords:** Brief intervention, Hazardous drinking, Text messaging, Pilot randomised controlled trial, India

## Abstract

**Objective:**

To evaluate the feasibility and acceptability of a mobile-based brief intervention (BI), generate preliminary estimates of the impact of the BI and fine-tune the procedures for a definitive randomised controlled trial.

**Design:**

Parallel three-arm single-blind individually randomised controlled pilot trial. Eligible and consenting participants were randomised to receive mobile-based BI, face-to-face BI and information leaflet.

**Setting:**

Educational institutions, workplaces and primary care centres.

**Participants:**

Adult hazardous drinkers.

**Results:**

Seventy-four participants were randomised into the three trial arms; forty-eight (64·9 %) completed outcome evaluation. There were no significant differences between the three arms on change in any of the drinking outcomes. There were however in two-way comparisons. Face-to-face BI and mobile BI were superior to active control for percent days heavy drinking at follow-up, and mobile BI was superior to active control for mean grams ethanol consumed per week at follow-up.

**Conclusion:**

The encouraging findings about feasibility and preliminary impact warrant a definitive trial of our intervention and if found to be effective, our intervention could be a potentially scalable first-line response to hazardous drinking in low-resource settings.

Alcohol use disorders (AUD) are major contributors to morbidity, mortality and disability across the world^(1)^. India is experiencing a steady increase in alcohol consumption, and increasing levels of alcohol-related problems^(2,3)^. Despite the higher prevalence of hazardous drinking (pattern of drinking that confers reasonably high risk of harm), compared to those already experiencing harm due to AUD, for example, dependent drinkers (20 % *v*. 4 %), Indian health policy focuses predominantly on the latter; resulting in the former not having access to adequate help^(3,4)^.

Brief interventions (BI) are short personalised, supportive and non-judgemental interventions delivered to help the individual to understand that their drinking is putting them at risk and to encourage them to reduce or give up their drinking. Although there is extensive evidence demonstrating the effectiveness of BI in reducing hazardous drinking^(5)^, the key barrier has been in the translation of the research evidence into implementation in routine practice^(6)^. Some of the challenges influencing the implementation of BI in routine practice in low- and middle-income countries (LMIC) include the shortage and inequitable distribution of health professionals, high clinical workload and competing clinical priorities^(7–11)^.

One evidence-based solution to make interventions accessible in low-resource settings is through the use of technology, thus addressing the key barrier related to shortage of human resources^(12)^. Although there is growing evidence that digital health interventions may lower alcohol consumption, all the studies have been conducted in high-income countries and the interventions are web-based and require computer literacy^(13)^; characteristics that limit the potential scalability of these interventions in LMIC.

On the other hand, the relatively less sophisticated short message services (SMS) and interactive voice response (IVR) can be used to reach large numbers quickly and at low cost, to overcome language and literacy barriers, and to access people in remote areas. SMS-based interventions have been tested on multiple therapeutic targets such as appointment attendance, motivation, self-efficacy, relapse prevention and social support in those with drug or alcohol dependence^(14)^, but the potential of such interventions for hazardous drinking has not been explored in LMIC^(15,16)^.

The aim of the Alcohol use disorders-Mobile based Brief Intervention Treatment (AMBIT) study was to develop and evaluate a mobile-based BI for people with hazardous drinking in India. The preliminary formative research efforts in this study informed the development of the first version of the treatment package^(17)^. This first version of the package was tested through a case series, by refining the intervention content and delivery mechanisms through an iterative process, to develop the final intervention (submitted for peer review). The pilot randomised controlled trial described in this paper is aimed to empirically evaluate the feasibility and acceptability of the mobile-based BI, generate preliminary estimates of the impact of the BI and fine-tune the procedures for a definitive randomised controlled trial.

## Methods

### Study setting

The study was conducted in Goa, a small state on the west coast of India. In Goan men, the prevalence of high-risk drinking in a community was 14·8 %, and that of hazardous drinking ranged from 15 % in samples drawn from primary care to 21·3 % in samples of industrial workers^(18–20)^. Recent evidence has also indicated increasing levels of alcohol consumption amongst young women in India^(21)^. The study was implemented in the following settings: educational institutions (two rural and five urban colleges), workplaces (thirteen police stations and four bus depots) and primary care (two community health centres).

### Design

Parallel three-arm single-blind individually randomised controlled trial. The trial protocol was registered on ClinicalTrials.gov (registration identifier NCT04078360).

### Study sample

A sample of 120 participants was planned to be recruited in the intervention and control groups, approximately evenly spread out across all study settings and interventions. The sample size was not based on formal power calculations as the study is not designed to test a hypothesis. The sample size estimations have been made based on our previous experiences of pilot randomised controlled trial and deemed sufficient to achieve the goals of understanding the acceptability, feasibility and preliminary impact of the intervention^(22)^. Recruitment commenced on 20 September 2019 and because of the COVID-19 pandemic we had to discontinue recruitment on 16 March 2020, before we achieved the target sample size.

The inclusion criteria were (a) adult (aged 18–65 years) males and females in educational institutions, or (b) adult males in workplaces and primary health centres (PHC), who were (c) hazardous drinkers defined as a score of 8–15 on the Alcohol Use Disorder Identification Test (AUDIT). The AUDIT is a 10-item screening questionnaire developed by the WHO for the detection of AUD^(23)^, and (d) had personal ownership of a mobile phone and familiarity with the use of SMS/IVR. The exclusion criteria were (a) females (in workplaces and PHC). There is insufficient evidence to indicate that hazardous drinking in women in India is as widespread across the lifespan as it is with men, so women older than the typical college-going age (25 years) were not included in the study and consequently were not screened at the workplace and primary care site, (b) harmful and dependent drinkers (AUDIT score >15), (c) individuals owning a shared/family phone and (d) those who expressed an inability to use SMS and IVR.

### Recruitment procedure

Participants across all three settings were recruited through screening by trained researchers, who identified hazardous drinkers through appropriate screening procedures. Eligible participants were recruited if they provided voluntary informed consent to participate.

To reduce stigmatisation associated with drinking, at educational institutions and workplaces, a ‘Health and Wellness Camp’ (tested during the formative research phase for acceptability and feasibility) was organised. In the screening room/booth set-up in the Camp, researchers screened for two other health-related issues (perceived stress and physical activity) in addition to AUD screening. At the PHC, where questions about drinking behaviours are not unusual, we did not expect similar stigmatisation and hence we directly conducted universal screening for hazardous drinking to identify eligible participants.

In addition, recruitment was also undertaken through referrals from the following sources: (a) self: potential participants who got to know about our study at all three settings through the various forms of advertising (e.g. banners, posters and leaflets) approached a researcher for screening at their respective and preferred location and (b) gatekeepers (e.g. faculty members/college staff/members of student bodies in colleges; human resource officials and peers in the workplace; and medical officers and other healthcare staff at the PHC): the researchers sought assistance from these gatekeepers to build awareness about the study and these gatekeepers referred potential participants to the researchers and informed people at the site about the health and wellness programme and asked them to make contact with the study team. Self-referring participants and those sign-posted by the gatekeepers were then screened using the procedures described.

### Screening

All potentially eligible participants were screened using the AUDIT to identify hazardous drinking. The AUDIT has been validated in India, used in cross-national studies and used extensively in the study setting^(24,25)^. It has been translated into Konkani (Goan vernacular), using a systematic translation-back translation method with two teams of translators, followed by an item-by-item analysis and selection by consensus^(20)^. Other screening tools that were used as part of the ‘Health and Wellness Camp’ activities included the International Physical Activity Questionnaire (IPAQ)^(26)^ and Perceived Stress Scale (PSS)^(27)^, both of which have been validated in LMIC. The PSS is one of the most widely used psychological instrument for measuring the perception of stress and has been empirically validated with populations of mainly college students or workers^(28)^. The IPAQ is a widely used questionnaire developed to collect health-related physical activity data and is suitable for use among young and middle-aged adults^(29)^. Participants screening positive for hazardous drinking as well as PSS or IPAQ were recruited into the study. Those who screened negative for hazardous drinking but positive on PSS and IPAQ were provided relevant information and advice about stress management and healthy physical activities. Those screening positive for harmful drinking or alcohol dependence on the AUDIT were provided relevant information and advice related to AUD, and information about relevant local services to access help.

### Baseline assessments

(a) Sociodemographic information such as age, gender, employment status, educational status and marital status; (b) Time-Line Follow Back (TLFB): the TLFB is a calendar method of collecting data about quantity and frequency of drinking and various memory aids are used to enhance recall (e.g. special dates)^(30)^. The TLFB has high test–retest reliability and concurrent validity has been established in various types of AUD. It has been used in previous research in the study settings^(25)^. (c) In addition, for the participants receiving the mobile-based intervention, we collected information required for the delivery of the intervention such as preferred days/time to receive the SMS/IVR, and preference for SMS and/or IVR.

### Randomisation

Stratified randomisation was used. Consenting eligible participants were randomised within the following three strata: educational institutions, workplaces and healthcare centres. Within each stratum, they were randomised to receive SMS/IVR-based BI, face-to-face BI or active control. An independent data manager generated a randomisation list and participants were randomised using Sequentially Numbered Opaque Sealed Envelopes to maximise allocation concealment. The research workers administering the outcome tools and the lead investigators were blind to arm allocation.

### Interventions

Mobile based-BI – the intervention included SMS or IVR calls twice or thrice a week delivered for a duration of 8 weeks. Once the participant’s mobile number was uploaded on our technology partner’s (https://viamo.io/) platform, messages/IVR calls were sent automatically to them as per schedule. Each week messages were focused on specific content areas derived from the formative research of the study. Some of the components of the intervention include self-awareness messages, self-reflection messages, motivational messages, messages on safe drinking, alcohol reduction, drinking management, risk management, craving management and drinking alternatives, health education messages, personalised feedback and information, and goal setting. Dropout from the intervention was defined as participants choosing to stop receiving the intervention messages by informing the project team. The messages were in English or the vernacular language based on the choice of the participants. The messages were predominantly ‘push’ messages (not requiring a response from the recipient) with a few ‘pull’ messages (requiring a response from recipient). Further details of the intervention are available in a separate peer-reviewed publication^(17)^.

Face-to-face BI – the BI was based on the WHO Mental Health Gap Action Programme (mhGAP) intervention and was delivered by a trained researcher. The BI was delivered over a single session lasting 5 to 10 min. This included sharing their AUDIT score and its interpretation with the participant, personalised feedback, providing information about safe drinking limits, potential risks to health and social wellbeing if drinking continued at the present level, collaborative exploration of potential benefits of reducing drinking, and strategies to reduce drinking.

Active control – this was a BI leaflet consisting of information on alcohol consumption, and tips to manage and reduce drinking.

### Outcomes

The primary outcome was change in percent days abstinent (PDA). Secondary outcomes include change in quantity of drinking (grams ethanol/week), change in patterns of drinking (percent days heavy drinking (PDHD)) and change in intensity of drinking (grams ethanol per drinking day). All these outcomes were calculated from the TLFB administered at 3 months post-randomisation and covered the 2 weeks preceding the outcome evaluation.

### Feasibility and acceptability

Feasibility and acceptability were assessed by examining data on the number and proportion of dropouts from treatment (participants choosing to stop receiving the intervention messages), reasons for refusal to participate, reasons for dropout, proportion of participants choosing SMS *v*. IVR, proportion of participants that listened to the complete IVR and proportion of participants with high engagement (response to more than 50 % of messages requiring a response). The feasibility and acceptability were also examined through a nested qualitative study, the findings of which will be presented in a separate publication.

### Process indicators

The following process data were also collected during the course of the study: number of participants screened for AUD in each setting; number and proportion of participants screened positive for hazardous drinking; number and proportion of those with hazardous drinking who were eligible for the pilot trial; number and proportion of participants consenting for the treatment; and number and proportion of participants completing outcome assessment

### Analysis

Baseline characteristics of individuals who consented and did not consent, participants who did and did not complete outcome assessments, baseline characteristics of participants in the intervention arms *v*. those in the control arm were all analysed as proportions or means as appropriate and compared using chi-square test (for proportions), *t*-test (for means of two groups) and ANOVA (for means of three groups) as appropriate. For an overall statistically significant one-way ANOVA result, the Tukey’s *post hoc* test was used to confirm the differences between two groups. The trial process indicators are presented consistent with CONSORT guidelines^(31)^ including a trial flow chart. The primary analysis was intention-to-treat at the 3-month end point regardless of treatment adherence and adjusted for baseline AUDIT and recruitment site. Linear regression was conducted for comparing the difference between arms on change in the various drinking outcomes, and effect sizes are reported as adjusted mean differences with 95 % CI. *Post hoc* analysis included linear regression to compare the difference between arms on the various drinking outcomes at follow-up.

## Results

**Fig. 1:**
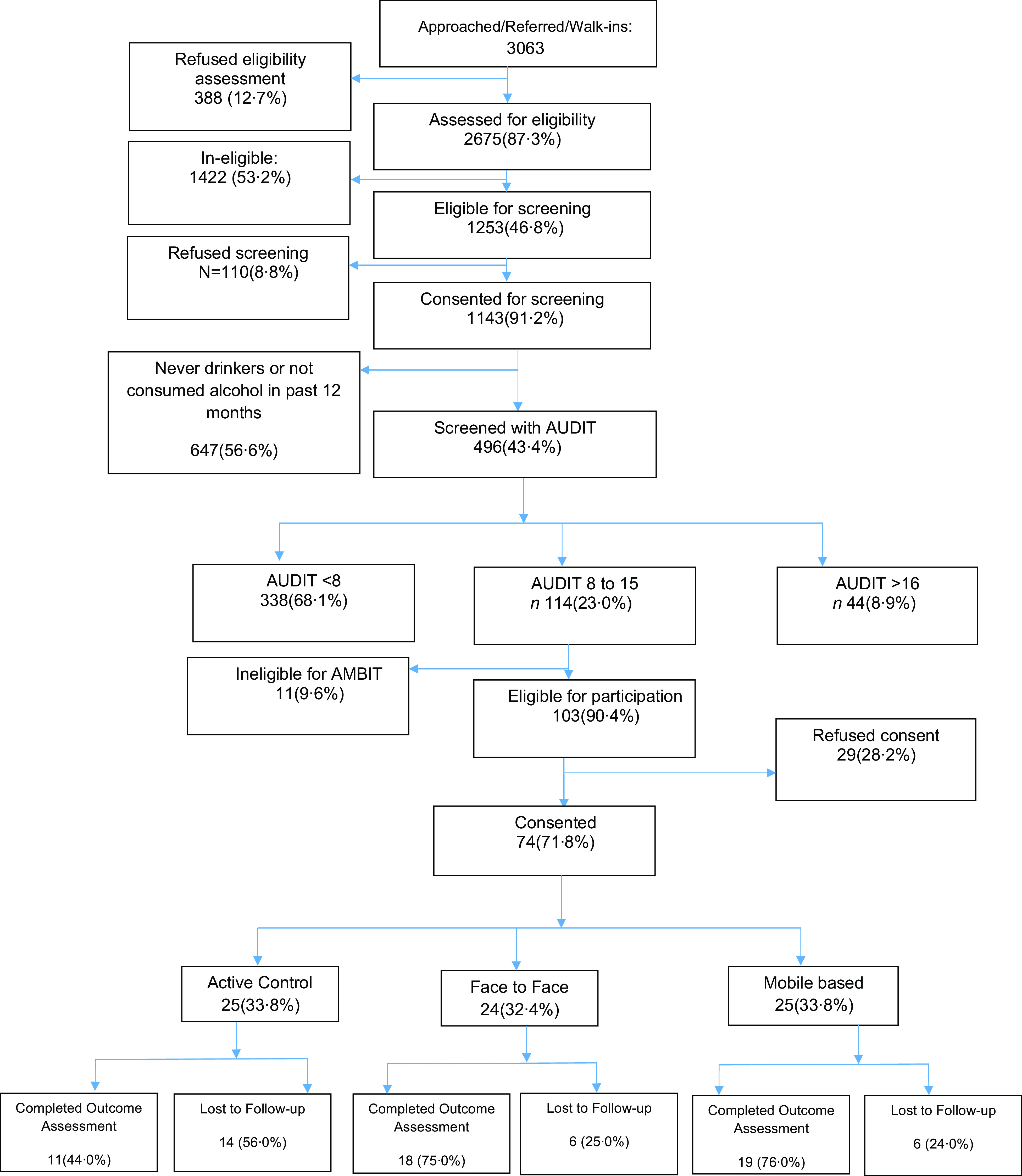
AMBIT RCT flow chart. AMBIT, Alcohol use disorders-Mobile based Brief Intervention Treatment; RCT, randomised controlled trial. AUDIT, Alcohol Use Disorder Identification Test

Figure 1 summarises the flow of participants through the trial. Reasons for refusing eligibility check for screening included not having time 177 (45·6 %), not being interested in the study 76 (19·6 %), worried about missing their position in the queue to consult the doctor 130 (33·5 %) and no reason given 5 (1·3 %). Reasons for ineligibility for screening included age less than 18 years or more than 65 years 172 (12·1 %), difficulty hearing/speaking 20 (1·4 %), did not understand English/vernacular 6 (0·4 %), not planning to be resident in the study area for next 6 months 300 (21·1 %), did not own mobile phone 9 (0·6 %), female in workplace/PHC 801 (56·3 %) and not able to respond to questions as they were in a critical condition 54 (3·8 %). Reasons for refusing screening included not having time 4 (3·6 %), not being interested in the study 102 (92·7 %), worried about missing position in the queue to consult the doctor 2 (1·8 %) and no reason 2 (1·8 %). The mean AUDIT score of those screened was 6·4 (sd 6·2) (range 0–32). Of the 114 hazardous drinkers, 103 (90·4 %) were eligible for participation and 11 were excluded because 8 did not know how to respond to the text messages sent to a mobile phone and/or had previously not responded to the text messages on a mobile phone and/or did not know how to use IVR calls, and 3 females were not recruited from educational institutions, as we had met the numbers for recruitment. Lower education, being employed and recruitment in PHC were significantly associated with refusal of consent to participate (Table 1).

**Table 1:** Variables associated with refusal of consent to participate

Variable	Total *n* 103		Refused consent *n* 29 (28·2 %)		Gave consent *n* 74 (71·8 %)		*P*
	*n*	%	*n*	%	*n*	%	
Age in years							0·13
Mean			36·4		32·3		
sd			11·3		12·5		
Gender							0·11
Female	6	5·8	0	0	6	100	
Male	97	94·2	29	29·9	68	70·1	
Marital status							0·05
Single	48	48·6	9	18·8	39	81·3	
Married	55	53·4	20	36·4	35	63·6	
Education status							0·014
Up to primary	8	7·8	5	62·5	3	37·5	
Secondary/higher secondary	73	70·9	15	20·6	58	79·5	
Graduate and above	22	21·4	9	40·9	13	59·1	
Employment status							0·03
Employed	67	65·1	22	32·8	45	67·2	
Student	32	31·1	5	15·6	27	84·4	
Retired	2	1·9	0	0	2	100	
Homemaker	2	1·9	2	100	0	0	
Recruitment site							0·002
PHC	18	17·5	11	61·1	7	38·9	
Workplace	53	51·5	13	24·5	40	75·5	
Educational institution	32	31·1	5	15·6	27	84·4	
AUDIT score							0·06
Mean	10·7		10·0		10·9		
sd	2·2		2·2		2·2		

AUDIT, Alcohol Use Disorder Identification Test.

There was no significant difference between the three arms on baseline sociodemographic variables and AUDIT score (Table 2). Twenty-six (35·1 %) participants were lost to follow-up, and none of the baseline variable were associated with dropping out from the outcome evaluation (Table 3). Twenty-five participants each were randomised into the active control and mobile-based BI arms and twenty-four were randomised into the face-to-face BI arm. In the mobile-based intervention arm, nineteen (76 %) opted to receive SMS and the rest opted for IVR. Of those who opted to receive IVR, 42 % participants listened to all the IVR messages. Of those who opted to receive SMS, 95 % participants responded to more than half of the pull messages which required a response. None from the mobile-based BI arm chose to drop out from the intervention. There were no serious adverse events reported in any of the three arms.

**Table 2:** Comparison of the three trial arms at baseline

Variable	Total *n* 74		Active control *n* 25 (33·8 %)		Face-to-face BI *n* 24 (32·4 %)		Mobile BI *n* 25 (33·8 %)		*P*
	*n*	%	*n*	%	*n*	%	*n*	%	
Age in years									0·64
Mean	32·3		30·7		32·1		34·0		
sd	12·5		10·9		11·9		14·7		
Female gender	6	8·1	2	8·0	2	8·3	2	8·0	1·0
Marital status									0·84
Single	39	52·7	14	56	13	54·2	12	48	
Married	35	47·3	11	44	11	45·8	13	52	
Education status									0·61
Up to primary	3	4·1	2	8	1	4·2	0	0	
Secondary/higher secondary	58	78·4	18	72	20	83·3	20	80	
Graduate and above	13	17·6	5	20·0	3	12·5	5	20·0	
Employment status									0·40
Employed	45	60·8	16	64	15	62·5	14	56	
Student	27	36·5	9	36	9	37·5	9	36	
Retired	2	2·7	0	0	0	0	2	8	
Recruitment site									0·99
PHC	7	9·5	3	12	2	8·3	2	8	
Workplace	40	54·1	13	52	13	54·2	14	56	
Educational institution	27	36·5	9	36	9	37·5	9	36	
AUDIT score									0·10
Mean	10·9		11·5		11·1		10·2		
sd	2·2		2·2		2·1		2·1		
PDA									0·39
Mean	81·9		76·9		84·5		84·3		
sd	22·3		25·6		18·8		21·9		
PDHD									0·42
Mean	9·5		11·4		11·3		5·7		
sd	17·4		21·8		17·5		11·3		
Grams ethanol per week									0·39
Mean	126·5		167·2		124·4		87·9		
sd	202·9		284·5		168·9		116·8		
Grams ethanol per drinking day									0·54
Mean	47·5		45·2		52·9		44·8		
sd	23·1		22·0		19·0		28·2		

AUDIT, Alcohol Use Disorder Identification Test; PDA, percent days abstinent; PDHD, percent days heavy drinking.

**Table 3:** Baseline variables associated with loss to follow-up

Variable	Lost to follow-up *n* 26 (35·1 %)		Completed outcome evaluation *n* 48 (64·9 %)		*P*
	*n*	%	*n*	%	
Age in years					0·83
Mean	31·8		32·5		
sd	13·8		12·0		
Gender					0·92
Female	2	33·3	4	66·7	
Male	24	35·3	44	64·7	
Marital status					0·26
Single	16	41·0	23	59·0	
Married	10	28·6	25	71·4	
Education status					0·93
Up to primary	1	33·3	2	66·7	
Secondary/higher secondary	21	36·2	37	63·8	
Graduate and above	4	30·8	9	69·2	
Employment status					0·86
Employed	15	33·3	30	66·7	
Student	10	37·0	17	63·0	
Retired	1	50·0	1	50·0	
Recruitment site					0·08
PHC	5	71·4	2	28·6	
Workplace	11	27·5	29	72·5	
Educational institution	10	37·0	17	63·0	
AUDIT score					0·6
Mean	11·1		10·8		
sd	2·0		2·3		

AUDIT, Alcohol Use Disorder Identification Test.

In the overall sample, comparing baseline and follow-up, there was a significant decrease in mean PDHD (10·1 (18·7) *v*. 2·1 (5·7), *P* = 0·008) and mean grams ethanol consumed per week (62·6 (112·3) *v*. 21·9 (45·2), *P* = 0·02) (Appendix A).

There were significant within-arm changes in both the BI arms, with no within-arm changes in the active control arm for any of the outcomes. In the face-to-face BI arm, there was within-arm reduction in mean PDHD (9·1 (12·9) *v*. 0·8 (3·4), *P* = 0·02). In the mobile BI arm, there was within-arm increase in mean PDA (86·5 (16·5) *v*. 98·1 (3·2), *P* = 0·009) and reduction in mean grams ethanol consumed per week (38·3 (51·3) *v*. 3·7 (8·0), *P* = 0·01) (Appendix B).

There were no significant differences between the arms on change in any of the drinking outcomes (Tables 4 and 5).

**Table 4:** Between arm differences on change in alcohol consumption

	Active control *n* 11		Face-to-face BI *n* 18		Mobile BI *n* 19		*P*
	Mean	sd	Mean	sd	Mean	sd	
Change in PDA	8·4	37·1	2·0	26·6	11·7	17·2	0·53
Change in PDHD	−11·0	35·8	−8·3	13·5	−6·0	12·9	0·81
Change in grams ethanol per week	−65·8	207·1	−31·8	79·4	−34·6	52·9	0·71
Change in grams ethanol per drinking day	−1·2	44·0	−13·7	19·5	−20·4	7·9	0·69

PDA, percent days abstinent; PDHD, percent days heavy drinking.

**Table 5:** Intervention effect* as adjusted mean difference (95 % CI) for change in drinking

	Active control *v*. face-to-face BI		*P*	Face-to-face BI *v*. mobile BI		*P*	Active control *v*. mobile BI		*P*
	AMD	95 % CI		AMD	95 % CI		AMD	95 % CI	
Mean change in PDA (sd)	−2·4	-26·6, 21·8	0·84	10·8	-4·8, 26·4	0·17	6·4	-15·4, 28·1	0·55
Mean change in PDHD (sd)	−8·0	-20·9, 4·8	0·21	1·2	-7·8, 10·1	0·79	0·5	-16·9, 17·9	0·95
Mean change in grams ethanol per week (sd)	−24·7	-96·9, 47·5	0·49	−8·8	-55·0, 37·3	0·70	8·6	-83·9, 101·2	0·85
Mean change in grams ethanol per drinking day (sd)	−30·8	-67·0, 5·5	0·09	−12·6	-51·3, 26·1	0·44	−46·2	-225·3, 132·8	0·38

AUDIT, Alcohol Use Disorder Identification Test; PDA, percent days abstinent; PDHD, percent days heavy drinking;

* Adjusted for site as a fixed effect and baseline AUDIT score.

Mean PDHD at follow-up was significantly lower in the face-to-face BI arm compared to the active control arm (7·1 (9·6) *v*. 0·8 (3·4), *P* = 0·006), and in mobile BI arm compared to the active control arm (7·1 (9·6) *v*. 0·4 (1·6), *P* = 0·003). The mean grams ethanol consumed per week at follow-up was significantly lower in the mobile BI arm compared to the active control arm (55·6 (70·9) *v*. 3·7 (8·0), *P* = 0·005) (Appendix C).

Face-to-face BI (AMD −7·0; 95 % CI (−12·4, −1·5); *P* = 0·01) and mobile BI (AMD −6·7; 95 % CI (−11·7, −1·7); *P* = 0·01) were superior to active control for PDHD at follow-up, and mobile BI was superior to active control (AMD −44·0; 95 % CI (−79·5, −8·4); *P* = 0·02) for mean grams ethanol consumed per week at follow-up (Appendix D).

## Discussion

Our pilot trial has demonstrated that it is feasible to identify and recruit people with hazardous drinking in clinical, educational and workplace settings in Goa, that it is feasible to deliver a basic mobile-based intervention to such individuals, that this is potentially acceptable to the target group, and there are preliminary indications that a SMS-based BI might be superior to usual care even if it is enhanced, and at least as impactful as a face-to-face BI.

The effectiveness of BI for hazardous drinking has been extensively researched and established^(5)^, but there is limited evidence for both effectiveness and systematic implementation of BI in LMIC^(32,33)^. Although the effectiveness evidence makes BI a highly competitive option for public health interventions to reduce alcohol-related harm, implementation at scale has been challenging for several reasons. These include lack of trained healthcare staff, high clinical workload of existing staff, reluctance of patients experiencing alcohol problems to disclose such problems with healthcare providers, concerns about patient confidentiality and privacy, stigma related to AUD, all leading to limited accessibility, acceptability and adherence to treatment^(10,34–37)^.

For the change in drinking outcomes in our trial, although the changes are in the right direction, there are no significant differences between the arms. On the other hand, for the outcomes at follow-up, both BI demonstrated effects with the mobile BI showing even stronger effects than did the face-to-face BI. Our findings suggest the potential applicability of mobile-based BI for the management of hazardous drinking in low-resource settings. More importantly a mobile-based BI is uniquely positioned to overcome some of the key challenges to scaling up evidence-based BI, especially in low-resource settings. By eliminating the need of a human resource to deliver the intervention, we automatically exclude supply side barriers such as lack of trained professionals and reluctance to engage because of limited clinical time. This also overcomes demand side barriers related to stigma and related concerns about confidentiality. Finally, such a technology-enabled intervention has the added advantage of standardising the content of the intervention delivered by eliminating the variability in quality that can occur when delivered by humans. For such a programme to be scaled up, the only infrastructural requirement is telecom and with 1·2 billion mobile phone subscriptions (and growing) and a tele-density of 87 %^(38)^ India has an unique opportunity to significantly increase the penetration and coverage of BI to counter the increasing prevalence of hazardous drinking in the country.

As expected for a pilot trial not powered to test effectiveness, there were few statistically significant interventions effects. However, for the outcomes related to overall amount of alcohol consumed and heavy drinking days, the face-to-face and mobile-based interventions had more favourable outcomes compared to the control arm. Besides the lack of power to examine effectiveness, our study had other limitations. The drinking outcomes were based on self-report data, which could lead to differential social desirability responses between trial arms. Although our testing of multiple hypotheses increases the chances of false-positives, it is not unusual to do so in pilot trials to identify and test appropriate outcome measures for a definitive trial.

Our findings do indicate that for a trial of BI targeting hazardous drinking, outcomes focused on quantity or intensity of drinking might be better suited than those that measure frequency of drinking. So, the following power calculations for a definitive trial are based on the mean change in grams ethanol per drinking day observed in this pilot trial. Assuming a mean change of −20·4 (sd = 7·9) in the mobile BI arm and −13·7 (sd = 19·5) in the face-to-face BI arm, Type I error = 0·05, and 90 % power, we will need to recruit 326 participants, allowing for 35 % loss to follow-up. Finally, the findings of the pilot trial indicate that we will need to make vigorous attempts to ensure that the dropout from outcome evaluation is as low as possible.

The evidence base for effectiveness of BI is predominantly derived from high-income countries and implementation of BI at scale is fraught with challenges. Thus, our intervention is unique, as it is designed to be delivered using basic mobile phone technology making it potentially scalable even in low-resource settings. Considering our feasibility findings, a definitive trial of our intervention is warranted. If effective, our intervention could be positioned as a first-line response to hazardous drinking in India and other similar LMIC.

## Supplementary material

For supplementary material accompanying this paper visit https://doi.org/10.1017/S1368980022000313. click here to view supplementary material
