# Supplementary material for: Brief intervention for hazardous drinking delivered using text messaging: a pilot randomised controlled trial from Goa, India
Source: Public Health Nutr. 2022 Feb 18;25(5):1365–74. doi: 10.1017/S1368980022000313 (PMC7612702; doi:10.1017/S1368980022000313)
Supplement: Supplementary file 1 [file S1368980022000313sup.zip › S1368980022000313sup002.docx]

**Appendix A: Overall change in drinking**

|  |  | **N=48** | **p** |
| --- | --- | --- | --- |
| **Mean PDA (SD)** | Pre | 83.5 (19.9) | 0.06 |
|  | Post | 90.8 (20.7) |  |
| **Mean PDHD (SD)** | Pre | 10.1 (18.7) | 0.008 |
|  | Post | 2.1 (5.7) |  |
| **Mean grams ethanol per week (SD)** | Pre | 62.6 (112.3) | 0.02 |
|  | Post | 21.9 (45.2) |  |
| **Mean grams ethanol per drinking day (SD)** | Pre | 48.8 (21.9) | 0.21 |
|  | Post | 38.6 (21.9) |  |

**Appendix B: Within arm changes in alcohol consumption**

|  |  | **Active control**  **N=11** | **p** | **Face-to-face BI**  **N=18** | **p** | **Mobile BI**  **N=19** | **p** |
| --- | --- | --- | --- | --- | --- | --- | --- |
| **Mean PDA (SD)** | Pre | 72.1 (30.0) | 0.47 | 87.3 (13.1) | 0.76 | 86.5 (16.5) | 0.009 |
|  | Post | 80.5 (30.0) |  | 89.3 (22.6) |  | 98.1 (3.2) |  |
| **Mean PDHD (SD)** | Pre | 18.2 (31.2) | 0.33 | 9.1 (12.9) | 0.02 | 6.4 (12.6) | 0.06 |
|  | Post | 7.1 (9.6) |  | 0.8 (3.4) |  | 0.4 (1.6) |  |
| **Mean grams ethanol per week (SD)** | Pre | 121.4 (203.8) | 0.32 | 52.4 (68.2) | 0.11 | 38.3 (51.3) | 0.01 |
|  | Post | 55.6 (70.9) |  | 20.6 (38.8) |  | 3.7 (8.0) |  |
| **Mean grams ethanol per drinking day (SD)** | Pre | 53.2 (31.0) | 0.95 | 47.1 (18.6) | 0.11 | 43.6 (11.8) | 0.17 |
|  | Post | 52.0 (30.2) |  | 33.4 (12.9) |  | 23.2 (4.0) |  |

**Appendix C: Between arm differences on alcohol consumption at follow up**

|  | Active control  N=11 | Face-to-face BI  N=18 | Mobile BI  N=19 | p |
| --- | --- | --- | --- | --- |
| Mean PDA (SD) | 80.5 (30.0) | 89.3 (22.6) | 98.1 (3.2) | 0.07 |
| Mean PDHD (SD) | 7.1 (9.6) | 0.8 (3.4) | 0.4 (1.6) | 0.002 |
| Mean grams ethanol per week (SD) | 55.6 (70.9) | 20.6 (38.8) | 3.7 (8.0) | 0.007 |
| Mean grams ethanol per drinking day (SD) | 54.3 (27.6) | 31.3 (13.3) | 28.0 (20.8) | 0.08 |

Mean PDHD: Active control vs Face-to-face BI p=0.006; Active control vs Mobile BI p=0.003; Face-to-face BI vs Mobile BI p=0.97

Mean grams ethanol per week: Active control vs Face-to-face BI p=0.08; Active control vs Mobile BI p=0.005; Face-to-face BI vs Mobile BI p=0.43

**Appendix D: Intervention effect* as adjusted mean difference (95% CI) for drinking at follow up**

|  | Active control vs Face to face BI | p | Face to face BI vs Mobile BI | p | Active control vs Mobile BI | p |
| --- | --- | --- | --- | --- | --- | --- |
| Mean PDA (SD) | 4.4 (-15.1-23.8) | 0.65 | 6.5 (-4.3-17.2) | 0.23 | 14.3 (-0.7-29.4) | 0.06 |
| Mean PDHD (SD) | -7.0 (-12.4- -1.5) | 0.01 | -0.3 (-2.2-1.5) | 0.70 | -6.7 (-11.7- -1.7) | 0.01 |
| Mean grams ethanol per week (SD) | -27.3 (-68.3-13.6) | 0.18 | -12.9 (-31.4-5.7) | 0.17 | -44.0 (-79.5- -8.4) | 0.02 |
| Mean grams ethanol per drinking day (SD) | -27.2 (-57.4-3.0) | 0.07 | -2.4 (-27.5-22.7) | 0.83 | -27.8 (-86.1-30.6) | 0.29 |

***** adjusted for site as a fixed effect and baseline AUDIT score

**Cohen’s d** A vs B: Mean PDHD 0.61 (0.04-1.18); A vs C: Mean PDHD 0.72 (0.15-1.29), Mean grams ethanol per week 0.81 (0.23-1.39
